# Tumor-cell HLA-DR expression as a potential biomarker of immunotherapy response in hepatocellular carcinoma

**DOI:** 10.3389/fonc.2025.1700181

**Published:** 2025-11-11

**Authors:** Xinping Wang, Ningning Mao

**Affiliations:** Infectious Diseases Department, Weifang People’s Hospital, Weifang, Shandong, China

**Keywords:** hepatocellular carcinoma, immunothearpy, HLA-DR, MHC class II, tumor microenvironment

## Introduction

Immune checkpoint blockade (ICB) has reshaped the treatment landscape of several malignancies, yet durable responses in hepatocellular carcinoma (HCC) remain limited. In a recent study published in Advanced Science, Chang et al. conducted a comprehensive multi-omics and histological analysis of hepatitis B virus–associated HCC, identifying HLA-DR^+^ tumor cells as a defining molecular and immunologic feature of this etiology (1). The authors demonstrated that tumor-cell HLA-DR expression correlates with immune checkpoint activation, PD-L1 co-expression, CD8^+^ T-cell recruitment and exhaustion, and unfavorable clinical outcomes. Although the study primarily established these relationships at the correlative level, the depth of evidence strongly suggests that tumor-intrinsic MHC-II expression represents an important immunologic axis that may influence tumor immunogenicity and responsiveness to immunotherapy. These findings extend the concept of antigen presentation beyond professional antigen-presenting cells and reveal a previously underappreciated layer of immune regulation in virally driven HCC.

## Tumor-intrinsic antigen presentation and the search for biomarkers in HCC

Despite the success of PD-1/PD-L1 inhibitors in several malignancies, only a subset of patients derives durable benefit (2). Existing biomarkers—such as PD-L1 expression, tumor mutational burden (TMB), and interferon-γ gene signatures—only partially capture the complexity of tumor-immune interactions. Moreover, tumor-intrinsic antigen presentation via MHC-I has been the primary focus of mechanistic and biomarker research, with tumor-cell MHC-II expression often considered an aberrant or rare occurrence (3). Chang et al. contribute to this field by showing that HLA-DR, a key human MHC-II molecule, is expressed on tumor cells in HBV-associated HCC and is linked to specific immunological features.

Emerging evidence from other cancer types supports the broader relevance of tumor-cell HLA-DR expression. For instance, a study in melanoma demonstrated that MHC-II expression represents a tumor-autonomous phenotype and predicts response to anti-PD-1/PD-L1 therapy (3). Similarly, high HLA-DR expression on tumor cells in laryngeal squamous cell carcinoma has been linked to better prognosis and potential response to anti-PD-1/PD-L1 therapy, possibly through increased CD4+ tumor-infiltrating lymphocytes (4). Additionally, a study in classic Hodgkin lymphoma demonstrated that MHC class II and PD-L1 expression predicted outcomes after PD-1 blockade, suggesting a role for MHC-II in immunotherapy response (5). These findings across different cancers suggest that tumor-cell MHC-II could have wider implications beyond HCC, though further validation is needed.

## Key findings: HLA-DR^+^ tumor cells associated with immune features in HBV-associated HCC

By integrating a large single-cell RNA sequencing (scRNA-seq) dataset of HCC (160 samples from 124 patients) with multiplex immunofluorescence and tissue microarrays (198 HCC specimens), Chang et al. identified HLA-DR+ tumor cells as a distinctive feature of HBV-associated HCC compared to hepatitis C virus (HCV)-associated and non-B non-C (NBNC) cases. These tumor cells, which express MHC-II molecules typically found on antigen-presenting cells, were correlated with elevated PD-L1 expression, increased CD8+ T-cell recruitment, and enhanced T-cell exhaustion phenotypes.

Trajectory analysis of CD8+ T cells revealed distinct differentiation pathways in HBV-associated HCC, characterized by enriched exhaustion and stem-like features. HLA-DR+ tumor cells were associated with an immunosuppressive TME, including heightened immune checkpoint activation in tumor-immune cell interactions. Clinically, high proportions of HLA-DR+ tumor cells were linked to poorer survival outcomes, particularly when co-expressed with PD-L1.

## Potential functional role: tumor-cell HLA-DR and T-cell interactions

While the study did not include direct functional experiments such as CRISPR-based models, the associations observed suggest that tumor-cell HLA-DR may contribute to T-cell engagement and modulation. For example, the enrichment of CD8+ T cells near HLA-DR+ tumor cells, coupled with increased exhaustion markers, implies a possible role in antigen presentation and immune suppression. This aligns with broader evidence from other studies where tumor MHC-II expression has been linked to CD4+ and CD8+ T-cell responses, However, it should be noted that these are correlations rather than demonstrated causative effects, and alternative explanations such as cytokine-driven induction (e.g., IFN-γ) of HLA-DR by infiltrating immune cells could also underlie the observed relationships.

## Implications: toward a biomarker for tumor immunogenicity in HCC

This study suggests a rationale for considering tumor-cell MHC-II expression—particularly HLA-DR—as a potential biomarker in HBV-associated HCC. Unlike current markers that reflect isolated aspects of the tumor-immune interface, HLA-DR+ tumor cells may integrate features such as antigen presentation, T-cell recruitment, and checkpoint activation. However, its predictive value for immunotherapy efficacy remains to be validated in prospective studies.

Clinically, assessing tumor HLA-DR status may help refine patient selection for ICB, especially in HBV-endemic regions, and guide combinations with therapies that enhance MHC-II expression or support T-cell function.

However, potential confounders must be considered to balance this perspective. For instance, HBV etiology itself may influence HLA-DR expression independently of immunotherapy response, and co-factors like PD-L1 levels, tumor stage, cirrhosis presence, or treatment regimens (e.g., monotherapy vs. combination) could affect outcomes. The retrospective nature of the immunotherapy cohort and limited sample size also introduce possible biases, such as selection effects or unmeasured variables. Neutral or conflicting findings from other HCC studies should be explored; for example, not all viral-associated HCCs may exhibit the same patterns, and NBNC cases showed different immune profiles in this analysis.

## Future directions: validating and targeting tumor MHC-II

The association between tumor-intrinsic MHC-II expression and immune cell activity highlights new opportunities for therapeutic development. Prospective clinical studies are essential to validate HLA-DR as a predictive biomarker for ICB efficacy, potentially incorporating multi-omics approaches to account for confounders. Therapeutic strategies could include agents that upregulate MHC-II (e.g., epigenetic modulators) combined with ICB to boost responses in low-HLA-DR tumors.

Furthermore, given the shared role of HLA-DR in both immune activation and autoimmunity, understanding the regulatory balance that permits tumor MHC-II expression without triggering off-target inflammation will be critical for clinical translation.

## Conclusion

Chang et al. provide evidence associating HLA-DR+ tumor cells with a distinct TME and potential immunotherapy responsiveness in HBV-associated HCC. While these findings suggest tumor-cell MHC-II as a promising node in the cancer immunity cycle, they remain hypothesis-generating and not yet indicative of a proven causal or predictive relationship.
